# Second booster dose improves antibody neutralization against BA.1, BA.5 and BQ.1.1 in individuals previously immunized with CoronaVac plus BNT162B2 booster protocol

**DOI:** 10.3389/fcimb.2024.1371695

**Published:** 2024-04-04

**Authors:** Guilherme R. F. Campos, Nathalie Bonatti Franco Almeida, Priscilla Soares Filgueiras, Camila Amormino Corsini, Sarah Vieira Contin Gomes, Daniel Alvim Pena de Miranda, Jéssica Vieira de Assis, Thaís Bárbara de Souza Silva, Pedro Augusto Alves, Gabriel da Rocha Fernandes, Jaquelline Germano de Oliveira, Paula Rahal, Rafaella Fortini Queiroz Grenfell, Maurício L. Nogueira

**Affiliations:** ^1^ Laboratório de Pesquisas em Virologia (LPV), Faculdade de Medicina de São José do Rio Preto (FAMERP), São José do Rio Preto, Brazil; ^2^ Diagnosis and Therapy of Infectious Diseases and Cancer, Oswaldo Cruz Foundation (Fiocruz), Belo Horizonte, Brazil; ^3^ Laboratório de Imunologia de Doenças Virais, Instituto Rene Rachou - Fundação Oswaldo Cruz, Belo Horizonte, Brazil; ^4^ Laboratório de Imunologia Celular e Molecular, instituto Rene Rachou - Fundação Oswaldo Cruz, Belo Horizonte, Brazil; ^5^ Laboratório de Estudos Genômicos, Departamento de Biologia, Instituto de Biociências Letras e Ciências Exatas (IBILCE), Universidade Estadual Paulista (Unesp), São José do Rio Preto, Brazil; ^6^ Department of Infectious Diseases, College of Veterinary Medicine, University of Georgia, Athens, GA, United States; ^7^ Hospital de Base, São José do Rio Preto, Brazil; ^8^ Department of Pathology, University of Texas Medical Branch, Galveston, TX, United States

**Keywords:** SARS-CoV-2, vaccination, booster, omicron, variants, antibody neutralization

## Abstract

**Introduction:**

SARS-CoV-2 vaccines production and distribution enabled the return to normalcy worldwide, but it was not fast enough to avoid the emergence of variants capable of evading immune response induced by prior infections and vaccination. This study evaluated, against Omicron sublineages BA.1, BA.5 and BQ.1.1, the antibody response of a cohort vaccinated with a two doses CoronaVac protocol and followed by two heterologous booster doses.

**Methods:**

To assess vaccination effectiveness, serum samples were collected from 160 individuals, in 3 different time points (9, 12 and 18 months after CoronaVac protocol). For each time point, individuals were divided into 3 subgroups, based on the number of additional doses received (No booster, 1 booster and 2 boosters), and a viral microneutralization assay was performed to evaluate neutralization titers and seroconvertion rate.

**Results:**

The findings presented here show that, despite the first booster, at 9m time point, improved neutralization level against omicron ancestor BA.1 (133.1 to 663.3), this trend was significantly lower for BQ.1.1 and BA.5 (132.4 to 199.1, 63.2 to 100.2, respectively). However, at 18m time point, the administration of a second booster dose considerably improved the antibody neutralization, and this was observed not only against BA.1 (2361.5), but also against subvariants BQ.1.1 (726.1) and BA.5 (659.1). Additionally, our data showed that, after first booster, seroconvertion rate for BA.5 decayed over time (93.3% at 12m to 68.4% at 18m), but after the second booster, seroconvertion was completely recovered (95% at 18m).

**Discussion:**

Our study reinforces the concerns about immunity evasion of the SARS-CoV-2 omicron subvariants, where BA.5 and BQ.1.1 were less neutralized by vaccine induced antibodies than BA.1. On the other hand, the administration of a second booster significantly enhanced antibody neutralization capacity against these subvariants. It is likely that, as new SARS-CoV-2 subvariants continue to emerge, additional immunizations will be needed over time.

## 1 Introduction

The COVID-19 pandemic has highlighted the necessity of developing fast and effective control measures to avoid viral dispersion, disease severity, and health system collapse (Martins et al., 2023; Thakkar et al., 2023). These control procedures included varied approaches, from social distancing to the development of antivirals and vaccines for a long-term protection strategy (Golpour-Hamedani et al., 2023; Thakkar et al., 2023). In this scenario, many pharmacological interventions were proposed to improve immune coverage against SARS-CoV-2, resulting in different vaccines, with different mechanisms to induce immune response, being approved to the population (Zhou et al., 2022). This was a remarkable moment in the science history, since many efforts have been made to test and approve these vaccines in record time (Bravo et al., 2022; Falsey et al., 2021; Palacios et al., 2020; Sadoff et al., 2021).

Vaccination is the main process to provide mass immunity protection against infectious diseases, including viral infections, using safe mechanisms and approaches (Moradpour et al., 2023). The worldwide vaccination against SARS-CoV-2 played a crucial role on the control of the disease, reducing the number of cases, hospitalizations and deaths (Haas et al., 2022; Martinez-Baz et al., 2024; Steele et al., 2022). However, the process to produce and distribute the vaccines globally could not follow the rapid viral dispersion and evolution (Kupferschmidt, 2020; Lopez-Cortes et al., 2022). In fact, the delayed and gradual vaccination, associated with the high transmissibility and high mutation rate of the virus, contributed to the emergence and adaptation of SARS-CoV-2 variants capable to evade immune response induced by vaccination or prior infections (Mathieu et al., 2021; Rzymski et al., 2021).

Most of COVID-19 vaccines use Spike protein as the target of immunization action. The Spike protein is the viral envelope protein recognized by cell receptors during viral adsorption and entry steps, and this recognition is mainly mediated by the Receptor Binding Domain (RBD) present in the Spike (Shang et al., 2020). However, some mutations, especially those in the RBD, are capable to confer a lower affinity between the viral protein and antibodies induced by vaccination, resulting in a neutralization decrease (Martin et al., 2021). The majority of SARS-CoV-2 variants of concern (VOCs), responsible for the highest infections peak during the pandemic, present mutations in this region, such as Delta and Omicron, and many studies have already demonstrated that these variants are less neutralized by serological immune response induced by vaccination (CDC, 2021; Hoffmann et al., 2022; Planas et al., 2021; Planas et al., 2022).

Several strategies have been developed to avoid this immune response evasion. Initially, one approach involved administering a supplementary dose of the same vaccine to those who had received complete vaccination according to any SARS-CoV-2 vaccine’s primary protocol. In general, this procedure presented an enhancement on the protection against these VOCs, but in a small proportion (Furukawa et al., 2022; Schultz et al., 2022; Wen et al., 2023). Since different immunization mechanisms have been approved (inactivated virus, attenuated viral vector, viral subunit and mRNA), an alternative to improve the immune response induction is the administration of a heterologous booster, using a vaccine different from the one used as the primary protocol, where the immune system could be stimulated in different ways, generating a more complete immune response (Hillus et al., 2021; Atmar et al., 2022). The results were widely positive, showing that the heterologous booster, independently of the immunization mechanisms, indeed presented a higher antibody and cellular response enhancement when compared to the homologous dose approach, especially against omicron (Perez-Then et al., 2022; Yoo et al., 2023).

The improved serological response, generated by the booster dose, showed to be an important tool to oppose and decelerate SARS-CoV-2 evolution and adaptation by reducing viral spread (Theparod et al., 2023). Even though, subvariants of omicron continued to emerge (Callaway, 2022; Callaway, 2023; Tegally et al., 2022; Wang and Cheng, 2022), and some concerns were raised regarding durability of protection and coverage against these new subvariants (Willett et al., 2024). Studies have shown that, even after a booster dose, humoral response tends to decay over time (Ozbay Kurt et al., 2022; Favresse et al., 2023). In addition, the scientific literature already exposed the potential evasion of these subvariants from immune response induced by vaccination and prior infections (Iketani et al., 2022; Wang Q. et al., 2022; Wang et al., 2023). The fact is that, as long as new subvariants continue to emerge, new immunization approaches will likely be needed, such as additional booster from time to time and updated vaccine technologies.

In Brazil, the immunization program started in January 2021 with a primary vaccination protocol with the virus inactivated vaccine CoronaVac, the first approved by the national regulatory agency (ANVISA, 2021a; Wu et al., 2021; Zhang et al., 2021). The course of the pandemic led to the approval of a first heterologous booster dose with the mRNA vaccine BNT162b2 during the huge omicron infection wave that started at the end of 2021 (ANVISA, 2021b; Lamarca et al., 2023). After this infection peak, a second booster dose with BNT162b2 or viral vector vaccines (ChAdOx1-S or Ad26.CoV2.S) was available to avoid immune escape and another wave of cases, but the adherence was not high as expected by the Brazilian health ministry (while more than 167 million people were fully vaccinated with the primary protocol, only 43 million people received the first and second booster doses) (accessed on January 14^th^, 2024) (Brasil, 2023). At the time, the panorama of omicron lineages was constantly changing, with many subvariants being introduced, highlighting BA.5 and BQ.1.1, the most frequent in the country at that moment (de Menezes et al., 2023; de Sousa et al., 2023).

In this scenario of many doubts, our study aimed to investigate the impact of the first and second booster doses, in a cohort vaccinated with CoronaVac primary protocol, against the omicron subvariants (BA.1, BA.5 and BQ.1.1) circulating in the country during 2022.

## 2 Methods

### 2.1 Cohort and vaccination groups

**Figure 1:**
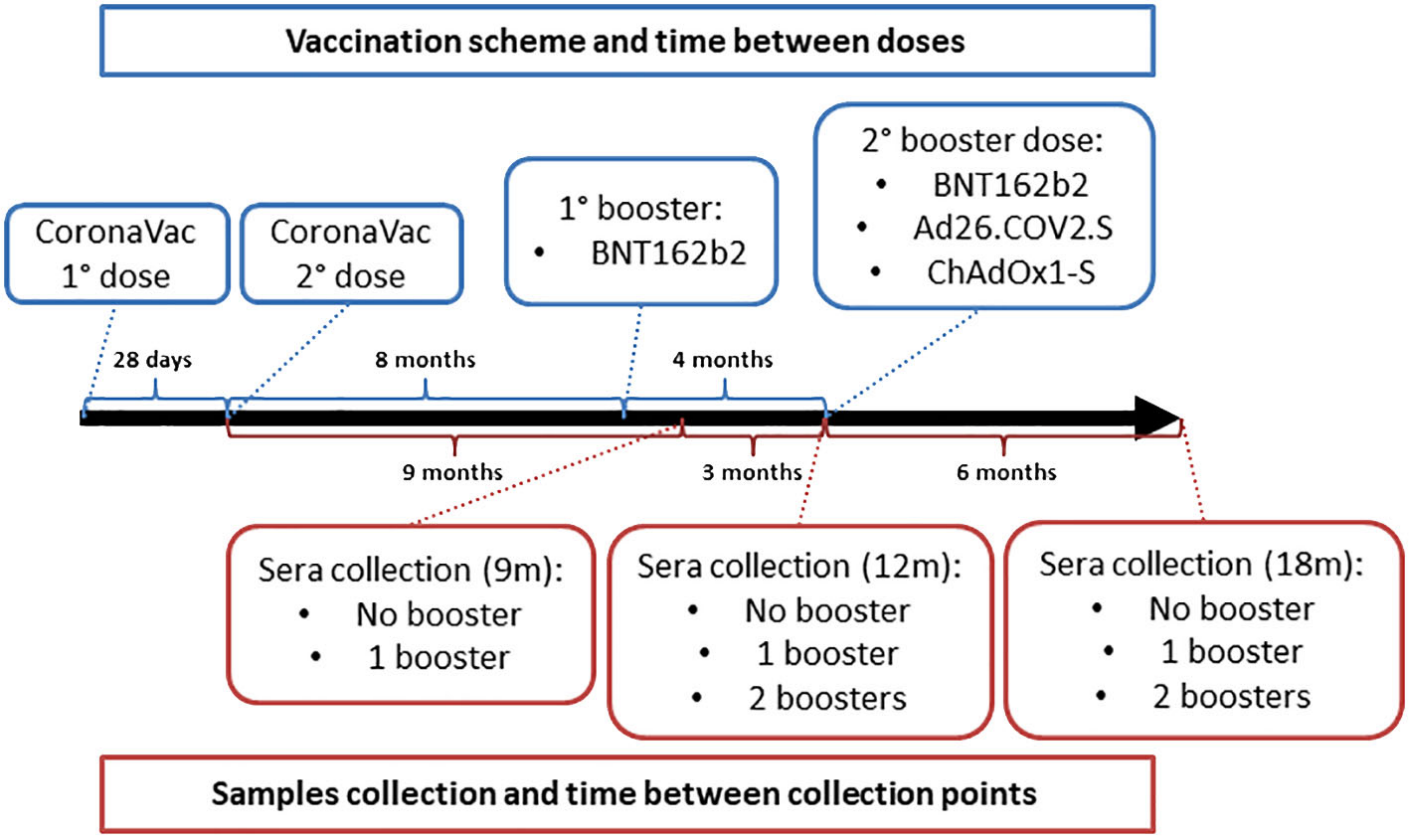
Schematic of vaccination and samples collection.

In order to analyze the induction of neutralizing antibodies against different sublineages of SARS-CoV-2 Omicron variant, serum samples were collected from individuals that received the primary protocol and were subsequently submitted to the booster doses over time. Samples were collected in three different time points after the CoronaVac initial protocol: 9 months (9m); 12 months (12m); and 18 months (18m). To assess the effectiveness of the booster doses, individuals were divided into three subgroups based on whether they had not received any additional doses (No booster), had received one additional dose (1 booster), or had received two additional doses (2 boosters), and all were monitored across the three time points (**Figure 1**).

For the first booster dose, the mRNA vaccine BNT162b2 was administered exclusively to the participants. Regarding the second booster dose, it was selected by the competent authorities the use of three different vaccines, BNT162b2 and the attenuated adenoviral vector vaccines Ad26.COV2.S and ChAdOx1-S. It is important to emphasize that bivalent vaccines were not used for this additional dose, only the first generation of vaccines were administered, and the choice of the vaccine was determined under availability. All the participants who were submitted to the booster doses were vaccinated at the same time, independent of the time-point group under analysis, which allows a fair comparison between the groups, subgroups and Omicron sublineages.

All individuals consented to be part of the study, which was approved by the institutional review board (IRB) of the Ethics Committee of the Oswaldo Cruz Foundation, under the protocol number CAAE 42898621.9.0000.5091.

### 2.2 Cell culture and SARS-CoV-2 omicron sublineages

For the *in vitro* tests, Vero cells (ATCC CCL-81) were cultivated in DMEM supplemented with 10% fetal bovine serum, 100 U/ml of penicillin and 100 µg/ml of streptomycin. Cells were maintained in a water-jacked incubator, at 37°C and 5% CO_2_.

To perform a comparison analysis on neutralization capacity, three SARS-CoV-2 Omicron sublineages were used in this study, the first Omicron variant introduced and isolated in Brazil, from sublineage BA.1, and two of the most recent sublineages that dominated the circulation in Brazil between 2022 and 2023, BQ.1.1 and BA.5. Sublineages BA.1 (HIAE –W.A) and BQ.1.1 (EPI_ISL_18277185) were isolated and kindly donated by Dr. Edison Durigon, from Universidade de São Paulo (USP), while BA.5 (EPI_ISL_18277186) was isolated in this study from a clinical sample. In this case, 300 µl of saline buffer from a nasopharyngeal swab sample was inoculated in Vero cells, at a confluence of 95%. Cells were incubated for 1h, for virus adsorption, and after this period, DMEM supplemented with 2% fetal bovine serum, 100 U/ml of penicillin and 100 µg/ml of streptomycin was added, followed by an incubation of 72h at 37°C. At this point, supernatant was collected and stored in a -80°C freezer until the neutralization tests. Before neutralization, virus was titrated by TCID_50_ (median tissue culture infectious dose), a method used do define the viral concentration necessary to infect 50% of the cultured cells, as previously described (Pastorino et al., 2020).

### 2.3 Viral microneutralization assay (VNT_50_)

The neutralization capacity of each sample, for each Omicron sublineage, was assessed independently by a viral microneutralization assay, as previously described (Campos et al., 2022). A total of 8 serum concentrations for each sample were tested, from 1:20 to 1:2560. After sera dilution, 50 TCID_50_/ml of virus was added to each well and incubated for 1h, at 37°C. After incubation, media from the cell plates was replaced by the mix containing virus and diluted sera, with a subsequent incubation at 37°C for 72h. Cell plates were fixed with 10% formaldehyde after incubation and stained with crystal violet. The absence of cytopathic effect is a result of viral neutralization, and the neutralization capacity was determined by the reciprocal dilution titer capable of inhibiting 50% of cytopathic effect in all replicates (VNT_50_).

The VNT_50_ calculation was performed using the Spearman-Karber algorithm (Spearman, 1908; Kärber, 1931). Seroconvertion, defined as the production and detection of neutralizing antibodies after vaccination with the primary protocol and the booster doses, was considered for all samples that presented VNT_50_ value over the cutoff of 20. This cutoff was defined according to the literature to maintain a stricter criterion, with higher specificity and avoiding false seroconvertion results (Favresse et al., 2021; Souza et al., 2021).

### 2.4 Statistical analysis

In order to compare the neutralization response of each omicron sublineage, in each time point and inside the vaccination groups, a two-way ANOVA was performed to evaluate the occurrence of any significant variance among the tested groups. After the variance analysis, the data from each time point was submitted to a Bonferroni’s multiple comparisons test, comparing to each other the VNT_50_ means of each sublineage, inside each vaccination group. For all analyses, the software GraphPad Prism 8 was used, and was considered statistically significant when the two-sided P value was lower than 0.05.

## 3 Results

### 3.1 Cohort samples collection

To evaluate the induction of neutralizing antibodies, individuals vaccinated with the two doses primary protocol of CoronaVac joined the study. Serum samples were collected 9 months (9m), 12 months (12m) and 18 months (18m) after the CoronaVac primary protocol. Inside each time point, samples were divided into three different subgroups according to vaccination schemes: no booster, 1 booster and 2 boosters, except for the 9m group, where the second booster was not available yet.

In total, 160 individuals were included in this study. For the 9m group, 41 samples were collected (15 for no booster; 26 for 1 booster). Regard the 12m group, 69 samples were obtained (20 for no booster; 30 for 1 booster; and 19 for 2 boosters); and for the 18m collection point, 50 samples were included (11 for no booster; 19 for 1 booster; and 20 for 2 boosters). The main characteristics of the cohort can be found in **Table 1**.

**Table 1:** General characteristics of the included participants.

	09 Months			12 Months				18 Months			
	No booster	1 booster	09m - ALL (n40) (N, %)	No booster	1 booster	2 boosters	12m - ALL (n68) (N, %)	No booster	1 booster	2 boosters	18m - ALL (n51) (N, %)
**Age** **18-30** **31-50** **51-62**											
	6 7 2	5 18 2	11 (27.5%) 25 (62.5%) 4 (10%)	2 16 2	6 13 11	4 13 1	12 (17.6%) 42 (61.8%) 14 (20.6%)	5 5 1	3 16 1	4 10 6	12 (23.5%) 31 (60.8%) 8 (15.7%)
**Gender** **Male** **Female**											
	6 9	6 19	12 (30%) 28 (70%)	8 12	8 22	4 14	20 (29.4%) 48 (70.6%)	4 7	1 19	6 14	11 (21.6%) 40 (78.4%)
**Comorbidities^a^** **Present** **Absent**											
	5 10	8 17	13 (32.5%) 27 (67.5%)	6 14	8 22	8 10	22 (32.3%) 46 (67.7%)	3 8	4 16	9 11	16 (31.4%) 35 (68.6%)
**Prior covid-19 infection^b^** **Yes** **No**											
	0 15	2 23	2 (5%) 38 (95%)	2 18	2 28	0 18	4 (5.9%) 64 (94.1%)	0 11	1 19	3 17	4 (7.8%) 47 (92.2%)
**Later covid-19 infection^c^** **Yes** **No**											
	6 9	10 15	16 (40%) 24 (60%)	8 12	8 22	13 5	29 (42.6%) 39 (57.4%)	6 5	6 14	7 13	19 (37.3%) 32 (62.7%)

a Comorbidities: Hypertension; obesity; diabetes; asthma; chronic kidney disease; hypothyroidism; dyslipidemia; chronic rhinitis; chronic sinusitis; rheumatoid arthritis; gastritis; endometriosis; sickle cell anemia.

b Infection before CoronaVac primary protocol.

c Infection after CoronaVac primary protocol.

### 3.2 Neutralization levels of BA.1, BA.5 and BQ.1.1 over time, with and without booster doses

The viral microneutralization assay allowed the evaluation of two parameters: the seroconvertion rates and the neutralization mean titers. The seroconvertion rates were determined by the number of individuals that presented a VNT_50_ value over the cutoff, and were represented by percentages. The neutralization mean titers were determined by the VNT_50_ mean of each group of samples tested against the omicron sublineages.

**Figure 2:**
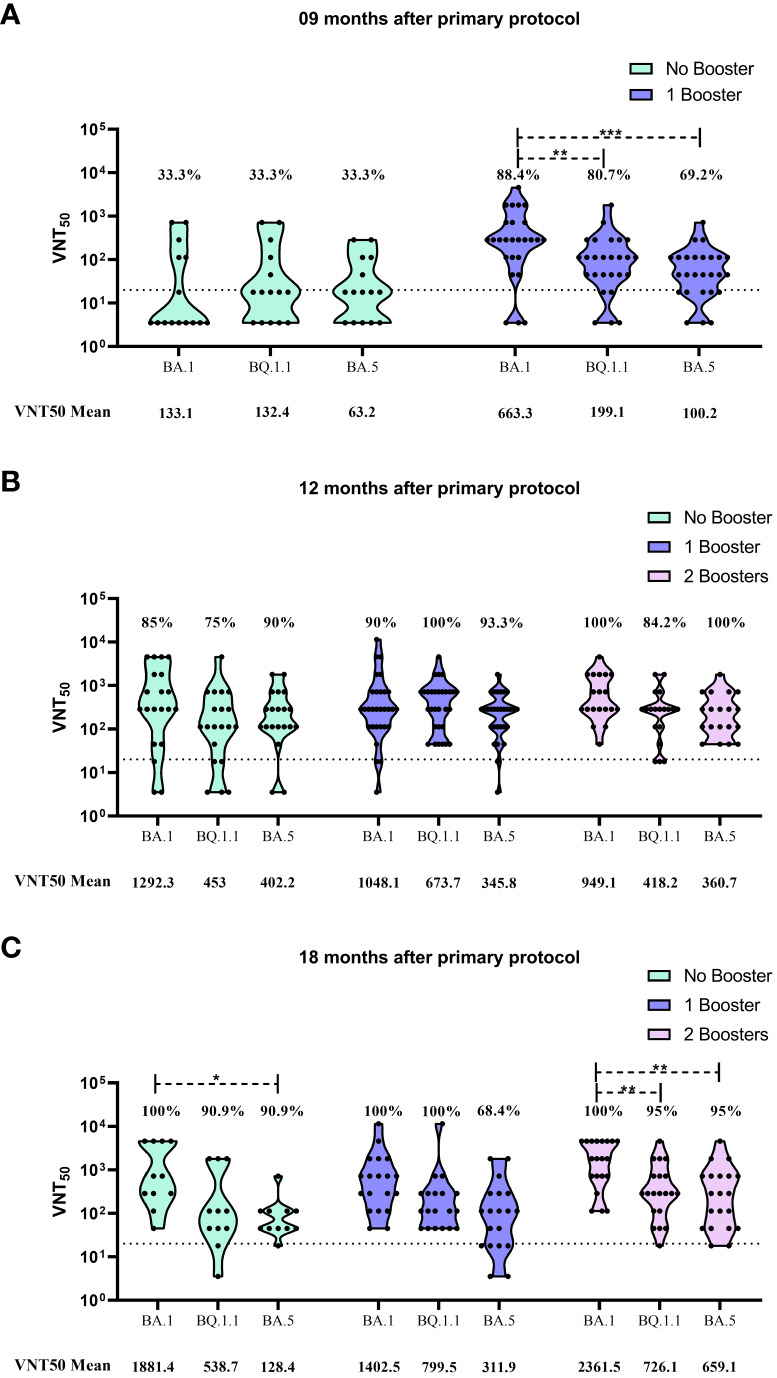
Viral microneutralization assay against omicron sublineages to evaluate neutralization titers (VNT_50_) and seroconvertion rate. **(A)** VNT_50_ for samples collected 9 months after CoronaVac 2 doses protocol. **(B)** VNT_50_ for samples collected 12 months after CoronaVac 2 doses protocol; **(C)** VNT_50_ for samples collected 18 months after CoronaVac 2 doses protocol. Vaccination subgroups are represented in green (No booster), blue (1 booster) and pink (2 boosters). VNT_50_ means, for each group, are highlighted under the graphs. Dashed lines represent the seroconvertion dilution cutoff (1:20), while seroconvertion rates are defined as percentages. The significance lines represent the differences among the mean neutralization titers of the groups. It was considered p-values lower than 0.05 for the significance. One asterisk (*) = p-value < 0.05; two asterisks (**) = p-value < 0.01; and three asterisks (***) = p-value < 0.001.

At the 9m time point, only the first booster was approved, and marked exactly one month after the administration of this additional dose. Thus, only two subgroups were evaluated at this moment: No booster and 1 booster. The findings here presented highlight that the first booster dose was capable to improve neutralization levels against all omicron sublineages. The VNT_50_ mean against BA.1 increased almost 5-fold, enhancing from 133.1 to 663.3. When compared to the neutralization improvement for BQ.1.1 and BA.5 (132.4 to 199.1 and 63.2 to 100.2, respectively), this two sublineages presented a significantly lower antibody neutralization induced by the first booster dose, when compared to BA.1 (p-values of 0.0042 and 0.0004, respectively). For seroconvertion rates, an improvement was observed for all sublineages after the booster dose (33.3% to 88.4%, 80.7% and 69.2% for BA.1, BQ.1.1 and BA.5, respectively) (**Figure 2A**).

The 12m collection time point marked the moment which the second booster was available and 4 months after the administration of the first booster. The data showed that the groups that received 1 or 2 boosters presented higher seroconvertion rates than those individuals that received only the CoronaVac primary vaccination. However, no difference was observed between the 1 booster and 2 booster groups at that moment. Regarding the neutralization titers, there was no difference when comparing the VNT_50_ mean values of each sublineage among the vaccination subgroups, but as observed for the 9m group, neutralization titers for BA.1 was higher than BQ.1.1 and BA.5 (**Figure 2B**).

When serum samples were collected at the 18m time point, 6 months had already passed since the second booster dose was applied, and 10 months after the first booster administration. The neutralization results showed that no significantly difference was observed in the seroconvertion rates among the vaccination subgroups. For all the tested sublineages, the number of seropositive individuals was similar independently of receiving booster doses or not (**Figure 2C**). An important observation is that, for BA.5, seroconvertion rate for the 1 booster subgroup decayed over time (93.3% at 12m to 68.4% at 18m). However, the second booster was capable to recover seroconvertion rate for BA.5, reaching 95% (**Figures 2B, C**).

On the other hand, when evaluating the neutralization titers, some significant differences were detected. In the no booster subgroup, the VNT_50_ mean for BA.1 was significantly higher than the observed for BA.5 (1881.4 and 128.4, respectively, with p-value of 0.0497). When observing the values for the 2 boosters group, it is noticeable that the neutralization levels were considerably improved for all sublineages when compared to the other vaccination subgroups (2361.5 for BA.1, 726.1 for BQ.1.1 and 659.1 for BA.5) (**Figure 2C**).

Despite this general improvement, the induction of neutralizing antibodies by the second booster was significantly higher for BA.1 when compared to the other omicron sublineages, being 3.25 and 3.58 fold higher than BQ.1.1 (p-value of 0.0099) and BA.5 (p-value of 0.0068), respectively (**Figure 2C**).

## 4 Discussion

The approval of the first SARS-CoV-2 vaccines, in the beginning of 2021, allowed the gradual return to normalcy worldwide. After a long time of social isolation, restrictions and uncertainty, the development and distribution of vaccines with different immune platforms enabled the so desired mass immunization.

Differently from most of the countries, the COVID-19 vaccination in Brazil was initiated with CoronaVac, and this vaccine was responsible to protect the most affected individuals at that moment (healthcare workers, elderly and people with comorbidities). This was essential to reduce COVID-19 dispersion, hospitalization and deaths during a critical period (Gamma circulation) (Wu et al., 2021; Banho et al., 2022).

The success of this immunization protocol may be related with the fact that CoronaVac utilizes an inactivated virus technology, comprising the whole SARS-CoV-2 viral particle as the immunizing agent (Gao et al., 2020). In general, inactivated vaccines are associated with higher safety levels and mild adverse events when compared to attenuated virus and viral vector vaccines (Murdin et al., 1996; Vellozzi et al., 2009; Xia et al., 2020; Ghattas et al., 2021). Additionally, when compared to mRNA, viral vector and subunit vaccines, known to induce the immune system using only a part of the virion, the use of a completely inactivated virus, with many viral epitopes, could allow the induction of a broad-spectrum immune response, with a wider efficacy range when compared to the other platforms, probably providing a greater range of protection against the first emerging variants of concern (Alpha, Gamma and Delta) (Can et al., 2022; Cerqueira-Silva et al., 2022; Grenfell et al., 2022).

Despite all the approved vaccines were efficient in the induction of both serological and cellular immune responses (Costa et al., 2022; Zhang et al., 2022), reducing number of cases, probability of disease progression and deaths, they were not able to inhibit completely the viral circulation, especially in those countries where vaccination programs were still delayed.

This phenomenon allowed, in a lower scale, the continuous process of viral dispersion and evolution, resulting in the emergence of SARS-CoV-2 variants and subvariants capable to escape immune response. It is believed that, despite the divergent evolution of these new variants, with advantageous adaptations over their ancestor lineages, a mutation convergence trend on many hotspots of their RBD exists. These convergent substitutions would be responsible to guarantee evasion from neutralizing antibodies without negative impact on infectivity or transmissibility. These data suggests that herd immunity would not be able to protect from future infections, and could act in the opposite way, as a selective pressure to the emergence of new resistant subvariants (Yeh and Contreras, 2021; Cao et al., 2023).

As a result of this process, some omicron subvariants continued to emerge worldwide, leading to the conduction of studies to evaluate the immune protection of the population and the possibility of new infection waves. Among these subvariants, BA.5 and BQ.1.1 were detected circulating in high frequencies in many countries.

It is believed that BA.5 was responsible for the initiation of the fifth infection wave of COVID-19 in the world, replacing BA.2 and becoming the most predominant subvariant in South Africa, USA and Europe as of June 2022 (Desingu and Nagarajan, 2022; Tegally et al., 2022). After this, in September 2022, BQ.1.1 emerged and became the most frequent variant around the world in January 2023, being responsible for more than 50% of the global cases (Ao et al., 2023; WHO, 2023). In Brazil, the same situation was observed, with these two subvariants being responsible for the majority of the cases in the same period (de Menezes et al., 2023; de Sousa et al., 2023).

The results presented here evidenced that both omicron subvariants BA.5 and BQ.1.1 are less neutralized by antibodies induced by vaccination, when compared to subvariant BA.1. This lower response can be observed in all vaccination groups (no booster, 1 booster and 2 boosters) and it is corroborated by the scientific literature (Wang XJ. et al., 2022; Tauzin et al., 2023). A study published by Cao et al, in June 2022, revealed that BA.5 was capable to escape serological immune response, showing increased capability to evade antibody neutralization when compared to other subvariants (Cao et al., 2022).

It is worth mentioning that this humoral response evasion was described for antibodies from a prior exposure to BA.1 and for vaccination induced antibodies (Cao et al., 2022). In this last situation, individuals were vaccinated with a 2 doses CoronaVac protocol, followed by a booster dose with CoronaVac or RBD protein subunit vaccine (ZF2001) six months later, showing the capacity of BA.5 to escape humoral response from homologous and heterologous booster schemes (Cao et al., 2022). Our results reinforce this reality, where one booster dose was not enough to enhance considerably the neutralization mean titers against BA.5, and this could be observed in all time points.

For BQ.1.1, a similar pattern was described by Planas et al. (2023), presenting capacity to evade neutralization from 6 therapeutic monoclonal antibodies and from sera of individuals vaccinated with a 2 doses BNT162b2 vaccination protocol, followed by a homologous booster shot (Planas et al., 2023). However, in our results, an improvement of neutralization mean titer (199.1 to 673.7) was observed from the 9m to the 12m time point after the first booster (**Figure 2B**), showing that the use of a heterologous approach was more responsive against BQ.1.1 than BA.5.

The use of additional boosters to improve immune response against some viral infections is widely recommended (Javanian et al., 2021; Pattyn et al., 2021). In some cases, there is no need to reformulate or update the vaccine booster, like the vaccination scheme against hepatitis B virus, for example (Pattyn et al., 2021). In other situations, where it is known that the virus can evolve quickly, the regular administration and update of booster doses is required, such as the annual vaccination against Influenza (Javanian et al., 2021). Since SARS-CoV-2 subvariants are continually emerging, administering additional booster doses from time to time will likely be a necessity.

Our data reinforces this hypothesis, where the administration of a second booster dose enhanced the antibody neutralization titers, against all tested omicron subvariants (especially against BA.1), 18m after the primary protocol and 6m after the second booster (**Figure 2C**). It is worth highlighting that, when compared to those individuals vaccinated only with one booster, in the same time point, neutralization titer against BA.5 doubled (311.9 to 659.1), showing that this approach could recover immune protection against immunoresistant subvariants.

The same fact was observed in a phase 2-3 study, where a second booster dose with mRNA-1273, after three doses of the same vaccine (homologous booster protocol), was able to recover the antibody neutralization response against BA.1 and BA.5. Additionally, the authors compared these results with a protocol using the updated vaccine mRNA-1273.214 (encoding both spike proteins from Wuhan ancestral lineage and BA.1) as the second booster, and it was observed that neutralization against BA.1 and BA.5 was even greater, suggesting that vaccines platform update will play a key role in the next rounds against SARS-CoV-2 (Chalkias et al., 2022; Chalkias et al., 2023).

Our study aimed to observe the impact of a second booster dose in a real life context, where people are coexisting, in constant contact with the recently emerged subvariants of omicron. In a situation like this, it is normal to observe the activation of memory immune response (Rodda et al., 2022), and this was present in our study, where seroconvertion rate and neutralization mean titers for the no booster group increased after 12m of the CoronaVac primary protocol.

At that moment, one year after the approval of the first COVID-19 vaccine, all of the restrictive measurements in Brazil were abrogated, and a peak of omicron BA.1 infection was on course, increasing exposure to the virus (Lopes et al., 2022; Lamarca et al., 2023). Despite the limitation imposed by this constant activation of memory immunity in the cohort, the results presented here clearly show that the second booster dose, when compared with no booster and 1 booster groups, 18m after the primary vaccination, improved considerably the antibody response against all subvariants (**Figure 2C**). This antibody response was robust and long-lasting, being detected after six months of the second booster, and was not noticed at the 12m time point (**Figure 2B**) probably due to the short period between second booster shot and serum collection.

For the conception of this study, some criteria regarding sample inclusion and time-point collection needed to be followed, and this resulted in some limitations. Samples from different individuals were randomly collected, for each time-point, in order to have a more representative and heterogeneous cohort, picturing a real-life scenario without any selection bias. Unfortunately, this approach does not allow an analysis over time of the same individuals, as a follow-up, which could enrich the data regarding the effectiveness of vaccination and boosters. Additionally, this decision led to a predominance of a female population in our cohort.

Other limitations were also present in our study. It was not possible to use of a single vaccine as the second booster, since vaccines for this specific situation were distributed according to availability at that moment, making it unfeasible to evaluate the effectiveness of a specific vaccine as a second booster shot. Another point is that, at the time period of the study, it was almost impossible to evaluate the impact of boosters isolated. As cited earlier here, most of the population is in constant exposure to other people and, consequently, to the virus. Despite not being able to evaluate the boosters in a controlled isolated environment, these limitations allowed an even closer analysis of a real-life situation, one of the aims of our study.

The findings presented here reinforce the concern regarding immunity evasion of the SARS-CoV-2 omicron subvariants, showing that BA.5 and BQ.1.1 are less neutralized by vaccine induced antibodies than their ancestor subvariant, BA.1. However, the use of a second booster dose, after a three doses protocol (two doses of CoronaVac followed by a first booster with BNT162b2) is capable to enhance and recover antibody neutralization against these subvariants. This may indicate that, as new subvariants continue to emerge, additional immunizations will be needed over time.

## Data availability statement

The raw data supporting the conclusions of this article will be made available by the authors, without undue reservation.

## Ethics statement

The studies involving humans were approved by the Institutional review board (IRB) of the Ethics Committee of the Oswaldo Cruz Foundation - Protocol number CAAE 42898621.9.0000.5091. The studies were conducted in accordance with the local legislation and institutional requirements. The participants provided their written informed consent to participate in this study. Ethical approval was not required for the studies on animals in accordance with the local legislation and institutional requirements because only commercially available established cell lines were used.

## Author contributions

GC: Conceptualization, Data curation, Formal analysis, Investigation, Methodology, Software, Validation, Visualization, Writing – original draft, Writing – review & editing. NA: Data curation, Investigation, Writing – review & editing. PF: Data curation, Investigation, Writing – review & editing. CC: Data curation, Investigation, Writing – review & editing. SG: Data curation, Investigation, Writing – review & editing. DM: Data curation, Investigation, Writing – review & editing. JA: Data curation, Investigation, Writing – review & editing. TS: Data curation, Investigation, Writing – review & editing. PA: Data curation, Investigation, Writing – review & editing. GF: Data curation, Investigation, Writing – review & editing. JD: Data curation, Investigation, Writing – review & editing. PR: Resources, Supervision, Writing – review & editing. RG: Conceptualization, Funding acquisition, Project administration, Resources, Supervision, Writing – original draft, Writing – review & editing. MN: Conceptualization, Funding acquisition, Project administration, Resources, Supervision, Writing – original draft, Writing – review & editing.
