# Supplementary material for: Second booster dose improves antibody neutralization against BA.1, BA.5 and BQ.1.1 in individuals previously immunized with CoronaVac plus BNT162B2 booster protocol
Source: Front Cell Infect Microbiol. 2024 Apr 4;14:1371695. doi: 10.3389/fcimb.2024.1371695 (PMC11024236; doi:10.3389/fcimb.2024.1371695)
Supplement: Supplementary file 1 [file DataSheet_1.pdf]

9 months after primary protocol				
	No Booster		1 Booster	
	Mean	SD	Mean	SD
**BA.1**	133,1873	249,7663	663,3862	997,494
**BQ.1.1**	132,446	248,8192	199,0308	362,4621
**BA.5**	63,21067	97,05909	100,2962	146,3179

12 months after primary protocol						
	No Booster		1 Booster		2 Boosters	
	Mean	SD	Mean	SD	Mean	SD
**BA.1**	1292,318	1753,456	1048,097	2280,32	949,1737	1110,99
**BQ.1.1**	453,0095	1002,356	673,7837	895,0462	418,2942	528,1699
**BA.5**	402,2465	531,3225	345,8817	364,0141	360,7611	431,193

12 months after primary protocol						
	No Booster		1 Booster		2 Boosters	
	Mean	SD	Mean	SD	Mean	SD
**BA.1**	1881,425	2134,922	1402,498	2670,038	2361,5	1922,286
**BQ.1.1**	538,7318	817,4712	799,5432	2597,871	726,077	1081,959
**BA.5**	128,4782	199,0219	311,9942	554,4435	659,1805	1064,767

Supplementary Table 1.VNT_{50} means and standard deviations against SARS-CoV-2 subvariants, for each vaccination and collection time point groups.
